# Plasticity in Colorectal Cancer: Why Cancer Cells Differentiate

**DOI:** 10.3390/cancers13040918

**Published:** 2021-02-22

**Authors:** Romina Judith Walter, Steffen Joachim Sonnentag, Véronique Orian-Rousseau, Leonel Munoz-Sagredo

**Affiliations:** 1 Karlsruhe Institute of Technology, Institute of Biological and Chemical Systems—Functional Molecular Systems, Hermann-von-Helmholtz-Platz 1, 76344 Eggenstein-Leopoldshafen, Germany; romina.walter@kit.edu (R.J.W.); steffen.sonnentag@kit.edu (S.J.S.); 2 Faculty of Medicine, University of Valparaiso, Angamos 655, Vina del Mar 2540064, Chile

**Keywords:** colorectal cancer, cancer stem cells, plasticity, cancer stem cell markers, tumor heterogeneity, tumor-organoids, lineage tracing, clonal cooperation, radio-resistance, serial transplantation, therapeutic resistance

## Abstract

**Simple Summary:**

The cancer stem cell hypothesis postulates that tumors arise from a few cells with self-renewal capabilities. The identification of stem cell markers, the development of mouse and human tumor organoids and their application in mouse models, allowing lineage tracing, helped to better understand the cancer stem cell model as well as the role of differentiation. This review aims at providing insights on the interplay between cancer stem cells and differentiated cells, as well as the importance of plasticity between the two states.

**Abstract:**

The cancer stem cell hypothesis poses that the bulk of differentiated cells are non-tumorigenic and only a subset of cells with self-renewal capabilities drive tumor initiation and progression. This means that differentiation could have a tumor-suppressive effect. Accumulating evidence shows, however, that in some solid tumors, like colorectal cancer, such a hierarchical organization is necessary. The identification of Lgr5 as a reliable marker of normal intestinal epithelial stem cells, together with strategies to trace cell lineages within tumors and the possibility to selectively ablate these cells, have proven the relevance of Lgr5^+^ cells for cancer progression. On the contrary, the role of Lgr5^−^ cells during this process remains largely unknown. In this review, we explore available evidence pointing towards possible selective advantages of cancer cells organized hierarchically and its resulting cell heterogeneity. Clear evidence of plasticity between cell states, in which loss of Lgr5^+^ cells can be replenished by dedifferentiation of Lgr5^−^ cells, shows that cell hierarchies could grant adaptive traits to tumors upon changing selective pressures, including those derived from anticancer therapy, as well as during tumor progression to metastasis.

## 1. Introduction

According to the cancer stem cell (CSC) hypothesis, only a small subset of cancer cells are capable of recapitulating the tumor of origin after serial transplantation into immunocompromised mice [1,2]. This distinguishes tumorigenic from non-tumorigenic cancer cells [3]. Profiling these operationally defined CSCs has enabled the identification of molecular markers that can be used to select cell populations with enhanced tumorigenic capabilities. This implies that tumors can be organized in cell hierarchies reminiscent of the ones in normal tissues, with stem cells at the apex, that give rise to transient amplifying progenitor cells that undergo processes of differentiation into several cell lineages. This hierarchical organization of cancer cells was initially proposed for acute myeloid leukemia [4], but has been extended to solid tumors, including colorectal carcinomas [5,6,7]. Initially proposed as an alternative to the model of clonal evolution of cancer cells [8] as a source of intra-tumoral heterogeneity, the concept of CSC and clonal evolution were found to be non-mutually exclusive, being now integrated with other sources of phenotypic variability like the ones influenced by distinct tumor microenvironments [2,9,10]. In parallel, the discovery of leucine-rich-repeats containing G-Protein-Coupled Receptor 5 (Lgr5) as a bonafide marker of normal intestinal epithelial stem cells [11] enabled an important step towards understanding stem cell properties and function in the intestinal epithelium. Direct lineage tracing showed that colorectal adenomas retain some of the hierarchical organization of their tissue of origin [12]. This experimental strategy—together with the development of ex vivo colorectal tumor organoids that are susceptible to further genetic manipulation, labeling, and transplantation—enabled researchers to trace the fate of mouse and human colon cancer clones in vivo during tumor progression and metastasis in mice [13,14,15,16]. It also enabled the selective ablation of Lgr5^+^ cancer cells in vivo, demonstrating their importance for colorectal cancer progression in animal models [13,14,15,16].

It is interesting to note, however, that from an evolutionary perspective, the hierarchical organization of cells in rapidly renewing tissues, like the intestinal epithelium during homeostasis, can act as a tumor suppressor mechanism, by minimizing cell divisions of stem cells, in charge of self-renewal, while depositing a higher proliferative burden on transit-amplifying cells destined to terminally differentiate into postmitotic cells that are shed out from the tissue. The fact that in colorectal tumors, cells expressing stem cell markers self-renew and give rise to cells expressing different markers of differentiated lineages [13,14,15,16,17], raises the question of the role of differentiated cancer cells along cancer progression. Does cancer cell differentiation confer any advantage to the tumoral ecosystem-primary or metastatic-subjected to Darwinian selective pressures? In this review, we explore available evidence to address this question. We conclude that a hierarchical organization of tumors, i.e., tumors containing cancer cells in different states along their process of differentiation, can play an important role in tumor progression. This does not refute the central role of CSCs and their relevance as targets for curative cancer therapy development, given that, as in the normal intestinal epithelium, in neoplastic lesions, Lgr5^+^ cells can be rapidly replenished by dedifferentiation of Lgr5^−^ cells after Lgr5^+^ cell damage or ablation [15,16]. This property, called cell plasticity, confers an adaptive trait to cancers, expanding their phenotypic repertoire to overcome selective challenges along cancer progression.

## 2. Cell Hierarchy Is a Tumor-Suppressor Mechanism

Cells in the human intestinal epithelium, which are exposed to the potentially harmful environment of the microbiome and digestive processes, are continually shed and replaced. This rapid cell renewal safeguards the physiological and structural function of this tissue: actively and passively absorbing nutrients while isolating other tissues from the processes of food degradation. However, high rates of cell division increase the likelihood of DNA-replication errors [18]. Many point mutations found in cancer cells arise from error-generating activities of DNA polymerases [19]. Although cells possess mechanisms to avoid genomic instability, when genes involved in these mechanisms are themselves the targets of mutations, a high mutagenic rate may ensue and lead to cancer. Indeed, families with hereditary non-polypous colorectal carcinoma harbor a germline mutation in genes required for post-replication mismatch repair that destabilizes their rapidly replicating genome [20].

**Figure 1:**
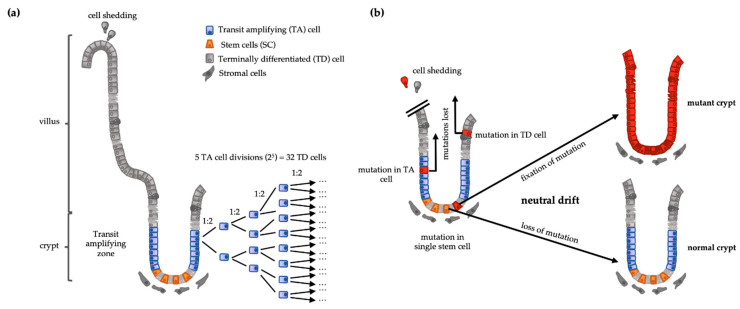
(**a**) Intestinal crypt-villus structure representing the stem cell (SC) function. If a stem cell generates a transit-amplifying (TA) cell and the latter divides 5 times, it will generate 32 (2^5^) terminally differentiated cells (TD) from a single stem cell division. (**b**) Intestinal cells harboring a mutation in a TA cell or a TD cell are shed and lost. Mutation in a SC gets fixed if the mutated SC dominates TD: Terminally differentiated; SC: stem cells; TA: Transit amplifying.

However, if cells engaged in rapid and repeated cell division are “dismissed from the task” of self-renewal, any mutation in these cells is lost when the mutated cell is lost, preventing its fixation in the tissue [21]. Terminally differentiated (TD) cells are post-mitotic and are shed into the intestinal lumen after a short-lived function. TD cells are generated from transit-amplifying (TA) cells that can undergo several cycles of symmetrical cell division before becoming TD cells. Because these cells do not self-renew, i.e., they all develop into TD cells, this compartment must be replenished by stem cells (SCs) which are in the cell compartment responsible for long-term self-renewal. Therefore, an eventual advantageous mutation in a cell of this compartment has a higher risk to remain within the tissue and be continuously transmitted to the clonal progeny thereafter. Conversely, since TA or TD cells do not self-renew, mutations occurring in them are likely to be lost upon cell shedding. If the cell division rate is directly proportional to mutation rate [18,19], it is beneficial to minimize the division rate of the self-renewing compartment and deposit cell number expansion in compartments of cells destined to be lost from the tissue. This three-compartment hierarchical structure of tissues—SCs, TA cells, and TD cells—enables to lower the division-rate self-renewal while rendering high numbers of TD cells, the product of an exponential expansion of TA cells. In homeostatic conditions, TA cells undergo 4–5 rounds of cell division [22], giving rise to 32 TD cells from a single SC division (Figure 1a). Using computational modelling of this system, Pepper and collaborators studied the impact of advantageous mutations—either increasing proliferation rates or decreasing cell death rates—arising in one of the three cell compartments. Unique mutations arising in the TA or TD compartments were always lost [21]. Thus, the location of the major proliferative function in a compartment devoid of self-renewal capacity allows high rates of cell turnover with low replicative mutagenic risk, resulting in a tumor-suppressor system.

However, although the SC compartment has a lower proliferative rate, most SCs divide daily [22]. If every SC would be constantly self-renewed—either by symmetric division into two daughter SCs or asymmetric division into a SC and TA cell—along the life span of an individual, they would undergo a number of divisions that would render a very high risk of mutations and they would be inevitably fixated in the tissue. This is indeed not the case at the level of individual SCs. Lineage tracing clearly revealed that the SCs compartment also undergoes a turnover following neutral competition dynamics. Without any intrinsic advantage, some SCs divide mostly symmetrically, displacing neighbor SCs out of the niche [22,23,24]. This means that mutations in the SC compartment can still be lost by neutral drift (Figure 1b).

Since differentiation can act as a tumor suppression mechanism by impeding self-renewal when a cell divides but does not further differentiate, it constitutes a self-renewing clone [25,26]. Indeed, when Pepper and collaborators introduced mutations that blocked cell differentiation to their model, these mutations were fixed in the tissue regardless of the compartment from which they arose [21]. This can explain the existence of undifferentiated tumors. However, it does not show how differentiated tumors arise within the constraints of this model.

Although transcriptomic analysis of in vivo colorectal cancer models shows that distinguishable cell compartments in CRCs are less defined—probably because of the loss of niche factor gradients that maintain them in non-neoplastic tissues—cells expressing stem cell markers are still distinguishable from populations expressing differentiation markers of diverse lineages reminiscent of normal tissues [13,15,16,17]. This means that differentiated tumors require that at least some cell-differentiation capabilities are retained. Therefore, if differentiation into non-tumorigenic cells is tumor-suppressive, why is this type of cell organization selected under the Darwinian mechanisms of cancer progression? One hypothesis is that for some tumors, such as differentiated colorectal cancers, establishment of different cell hierarchies through differentiation can be advantageous for the tumor. If this is the case, what are the selective advantages of such tumor organization? To assess this question, we will begin by analyzing the function of the putative CSCs as a baseline to distinguish the relevance of differentiated cells along tumor progression.

## 3. Cancer Stem Cells in Colorectal Tumors

### 3.1. Serial Transplantation and CSC Markers

CSCs have customarily been defined operationally: the ability to recapitulate tumors upon serial transplantation of cells from human cancers into immunocompromised mice. Recapitulation of the organization implies self-renewal capacities as well as potency of differentiation, reflecting the function of normal stem cells of adult tissues. The proportion of injected mice that developed disease was used to quantify the efficiency of tumor formation. Serial dilution of cancer cells decreased this efficiency, leading to the hypothesis that only a small subset of cells, from a heterogeneous bulk, possess these tumor-initiating capabilities. These findings set the conditions to search for cell surface markers to identify these tumorigenic cells. Fluorescent-activated cell sorting (FACS) enabled the enrichment of cell populations with cells possessing these markers, increasing tumorigenic efficiency with reduced numbers of injected cells. In the field-foundational study, cells from acute myeloid leukemia (AML) patients were injected into non-obese diabetic mice with severe combined immunodeficiency disease (NOD/SCID). The leukemia-initiating cells could be selected with the same markers (CD34^+^/CD38^−^) as normal hematopoietic cells that are able to repopulate NOD/SCID mice upon bone marrow transplantation [4]. This approach was subsequently extended to solid tumors like breast cancer [27], brain tumors [28], colorectal cancer [5,6,7], pancreatic cancer [29], and ovarian cancer [30]. 

CSCs of solid malignancies, as defined above, have been controversial [3,9,31]. Residual immune response to xenografts in immunodeficient mice might affect tumor initiation capabilities of cells. For example, although the melanoma tumor-initiating capacity was estimated to be present in only one of every million cells in NOD/SCID mice, transplantation into NOD/SCID IL-2Rγ-null (NSG) mice enabled tumor initiation from single cells in up to 30% of the animals [32]. This suggested that the assay was actually selecting cells with immune-evasive capabilities rather than reflecting the existence of a small stem cell pool. Instead, residual immune response could underestimate the putative CSC frequency and bias their marker profile. In addition, removal of cancer cells from their microenvironment in solid tumors by enzymatic dissociation and sorting could affect their fitness and tumor-initiation capabilities. This could also be the case for microenvironments that do not reflect the conditions in the tissue of origin, especially for heterotopic transplantation, thus compromising the tumorigenicity of clones that would otherwise possess it. Therefore, transplantation assays show the ability to form tumors despite these barriers and do not necessarily reflect the fates of cells in their tumor of origin. However, syngeneic serial transplantation assays have supported the hierarchical organization for mouse tumors, rejecting the possibility that these assays only reflect the action of clonal selective pressures, unrelated to a putative CSC function [3].

**Figure 2:**
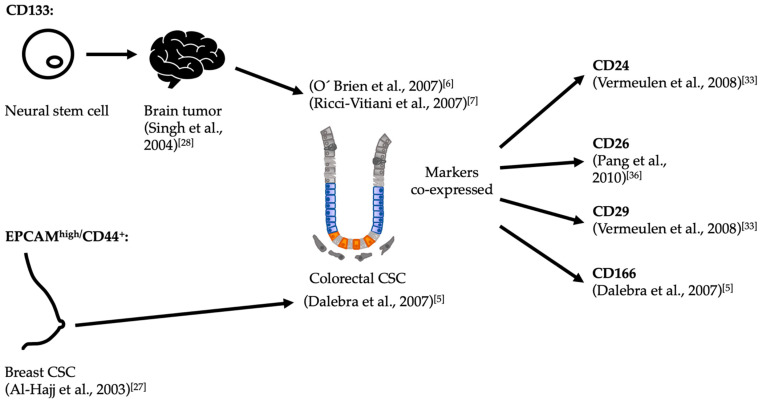
Historical development of CSC markers for colorectal cancer. In papers published back-to-back, O’Brien as well as Ricci-Vitiani and colleagues identified CD133^+^ colorectal cells as possessing tumor initiation capabilities upon heterotopic xenografts [6,7]. The criterion to probe this marker was derived from a previous experience with brain tumors, which in turn had derived from its expression in neural stem cells [28]. In parallel, Dalebra and collaborators had similar results, using the combination of the markers EpCAM^high^/CD44^+^ in subcutaneous injections [5,36]. In their case, the criterion to probe these markers derived from their previous work identifying putative CSCs from breast tumors [27]. Since none of these markers are expressed solely in putative CSCs, other associated markers were identified in an attempt to increase the specificity of markers for CSCs upon use in combination CSC: Cancer stem cell.

In spite of the limitations of serial transplantation, this method yielded several markers with the potential to identify cells with self-renewal and differentiation capabilities in vivo. The first marker identified for colorectal CSCs was CD133 [6,7]. In parallel, cells expressing high levels of EpCAM (epithelial cell adhesion molecule) while also expressing CD44 were also shown to enrich for tumor-initiating capabilities [5]. This discrepancy epitomizes the historical development of CSC markers, which were initially extrapolated either from markers known for normal stem cells or from the experience with models of other cancer types (Figure 2). However, further technical development supported their utility. Vermeulen and collaborators used single-cell cloning of CD133^+^ cells from patient samples to show that these cells could give rise to multilineage differentiated tumors upon implantation [33]. However, frequent loss of the epitope of CD133 during immunohistochemistry of patient histological samples prevented further clinical validation [34,35].

Refinement of the specificity of putative CSC markers can potentially be achieved by considering alternative splicing [37]. This can be specifically triggered by pathological processes like inflammation and cancer [38]. This may be the case for CD44. Notably, this adhesion molecule has been shown to be a putative CSC marker not only for colon cancer, but also for cancers of the bladder, breast, gut, head and neck, liver, lung, ovary, pancreas, prostate, and other organs [39]. However, CD44 is subject to complex alternative splicing that generates a myriad of isoforms in the extracellular domain. Consequently, multiple combinations of 9 variant exons in humans (10 in mice) and additional post-transcriptional modifications lead to high heterogeneity. Relevant to colorectal cancer, Todaro and collaborators showed that CD44v6—the CD44 family member most associated with malignancies—marks a population of cells that is highly tumorigenic and has metastatic potential [40].

Subsequent development of surface markers focused on those that reflect the function of signaling pathways known to be essential for stemness. In terms of activated signaling pathways, strong parallels were shown to exist between physiological and pathological conditions in the intestine [17,41]. Wnt signaling plays a fundamental role in homeostasis of the intestine, and malignant transformation is initiated by mutations in proteins of this pathway [42,43,44]. Wnt pathway mutations were detected in 92% of all colon cancer patients, 80% of which had a mutation in the *APC* gene [45,46,47]. This mutation leads to permanent activation of the canonical Wnt signaling pathway due to constant β-catenin stabilization followed by its nuclear translocation [43]. However, even if the majority of colorectal tumors have a hyperactivated Wnt signaling pathway, immunohistochemical studies showed that not every cell in a tumor exhibits this high Wnt activity [48]. Colorectal cancer cells with tumorigenic capacities were shown to exhibit high Wnt activity [33]. These cells upregulated stem cell markers, like Lgr5 and Ascl2, and recapitulated the Wnt activity heterogeneity upon tumor growth [49]. The advent of methods to trace the fate of stem cells in vivo [11] enabled the robust validation of one of these Wnt target-gene markers, Lgr5, as a marker of normal intestinal stem cells. The application of these methods to colorectal cancer, together with methods to precisely ablate Lgr5^+^ cells in vivo [50], initiated a new era for the CSCs concept, releasing it from its dependency on serial transplantation as an operational definition, and leading to deep insights into the function of these cells in cancer progression.

Although Lgr5 has been proven to be a bona fide marker of CRC-CSC and can be reliably used in available CRC in in vivo and ex vivo models, there is evidence of CRC not expressing Lgr5 [15,17]. This could be due to epigenetic silencing, while keeping high Wnt signaling [51] or by emergence of cells that are recognizable by alternative markers. Recent evidence from the Stappenbeck group identified Hopx as the marker of colitis-associated regenerative stem cells [52]. With chronic inflammation being a risk factor for the development of CRC, these findings could be relevant for some neoplastic processes.

### 3.2. Lineage Tracing and Organoids: Evidence for Cancer Stem Cells

Groundbreaking experiments with genetic lineage tracing were key to show that the crypt base columnar (CBC) cells at the bottom of the intestinal crypts function as bona fide stem cells [11]. This method allowed the identification of the Wnt target gene Lgr5, which is specifically expressed in the CBC cells, as a reliable intestinal stem cell (ISC) marker [11]. Barker and colleagues used the *Cre-loxP* genetic recombination systems to perform lineage tracing. In the *Lgr5-EGFP-IRES-CreER^T2^* mouse model, the Cre recombinase is expressed under the control of the cell-specific *Lgr5* promoter. This mouse line was crossed with *R26R–lacZ* reporter mice containing a *loxP-STOP-loxP* sequence in front of the reporter gene lacZ. The activated recombinase specifically activates the reporter gene expression in cells expressing Lgr5 by excising the *STOP* sequence. After the *STOP* sequence is removed, future descendent cells of the LacZ^+^ stem cells continue to express the reporter LacZ [11]. Both stem cell requirements were thus met by Lgr5^+^ CBC cells: the generation of multiple lineages and long-term self-renewal. Although this tracing cannot be performed in humans, stem cell dynamics have been successfully studied in the human colon when observing the spread of somatic mutations [53,54,55].

### 3.3. Tumor Organoids

The ex vivo organoids culture was an important development that enabled the further investigation of stem cell functionality [56]. Upon incorporation into a three-dimensional (3D) matrix, it was possible to grow single Lgr5^+^ adult stem cells, or crypts containing the stem cells isolated from the intestine, into three-dimensional multicellular tissue models. These organoids are maintained by the self-renewal of the intestinal stem cells, thus mimicking the in vivo properties of the intestine and remaining genetically and physically stable. The essential role of Lgr5^+^ stem cells in forming organoids was demonstrated using the *Lgr5DTR-eGFP* mouse model. In this model, Lgr5^+^ stem cells express eGFP and the diphtheria toxin receptor (DTR), which enabled only the stem cells to be marked and ablated. Lgr5DTR-eGFP tumor organoids treated with the toxin collapsed after losing their stem cells and were unable to regrow upon toxin treatment maintenance, thus demonstrating the necessity of Lgr5^+^ stem cells [16,50].

Methods developed to culture organoids originating from human adult colonic tissue [57] or human pluripotent stem cells [58] created the foundations to expand this technique to patient-derived tumor tissue. Lgr5^+^ stem cells were shown to be required for the formation of normal tissue organoids as well as tumor organoids [50]. The tumor organoids, however, differ phenotypically from the organoids originated from the healthy tissue. Relevantly, transcriptomic profiling of colorectal tumor biopsies and organoids derived from them showed that they conserve gene expression signatures from the tumor of origin [14].

Cultivation over long periods of time led to the establishment of biobanks containing a large number of differently characterized tumor organoids [59,60,61]. Furthermore, protocols to manipulate organoids by means of CRISPR/Cas9 [62,63] enabled modelling of common genetic mutations of the adenoma-carcinoma sequence of colorectal cancer in mouse organoids [13,16] and in organoids from human intestinal epithelium [64]. The same approach allows labelling of human tumor organoids from patient samples while simultaneously introducing transgenes that enable inducible and selective ablation of Lgr5^+^ cells [14,15]. Subsequent implantation of these engineered tumor organoids into mice led to lineage tracing and ablation experiments to study the role of Lgr5^+^ cells or other cell types of interest during tumor progression and metastasis.

The influence of stem cells and the result of their elimination in tumor tissue was also demonstrated with the Lgr5-eGFP-DTR transgenic mouse model [13]. In heterotopic tumors generated by subcutaneous injection of tumor organoids driven by APC, Kras, Trp53, and Smad4 mutations and carrying the Lgr5-eGFP-DTR allele, addition of diphtheria toxin (DT) efficiently eliminated Lgr5^+^ cells and resulted in restriction of tumor growth, although not in tumor regression. After the withdrawal of DT, the tumor was repopulated by Lgr5^+^ cells, reinitiating tumor growth. These experiments resulted in two important findings: (1) tumor development in this model of colorectal cancer, which is genetically relevant to the most common human colon cancer subtype, was driven by Lgr5^+^ cancer stem cells, and (2) tumors could be maintained by proliferative Lgr5^−^ cells that were able to replenish the eliminated Lgr5^+^ cells. In a spontaneous metastasis model derived from orthotopic tumors, the ablation of Lgr5^+^ cells did not provoke regression of the primary tumor but abrogated the formation of metastases in the liver. In contrast, metastasis developed rapidly in control animals. Ablation of Lgr5^+^ cells in experimental liver metastasis after injection of tumor organoids into the portal vein also ablated the metastatic tumor burden to barely detectable levels [13]. 

The lack of reliable antibodies against Lgr5 had previously hampered the study of Lgr5^+^ cells in human samples [14]. To engineer a human model to trace the role of Lgr5^+^ cells, Shimokawa and colleagues used CRISPR/Cas9-mediated homologous recombination to establish Lgr5-GFP clones or KRT20-GFP clones—marking differentiated cells—of colorectal cancer organoids derived from human colorectal tumors. Transcriptional analysis showed that Lgr5^+^ cells were enriched for intestinal stem cell markers, while Lgr5^−^ cells had upregulation of differentiation markers. Xenotransplantation of these organoids on the sub-renal capsule of NOG mice showed that the histological structure recapitulated the original tumor tissue structures. They found Lgr5-GFP^+^ cells in the outer area of the tumors. Complementarily, the KRT20-GFP^+^ differentiated cells were located in the inner area [15]. They established a lineage tracing strategy, using a multi-color rainbow reporter under control of *CreERT2* recombinase inserted into the *Lgr5* allele. Tamoxifen treatment at 1 month after xenotransplantation into the sub-renal capsule and strict temporal follow-up for 31 more days enabled them to reconstruct the clonal trajectories. Shortly after tamoxifen injection, single Lgr5^+^ clones appeared colored in the outer regions, followed by structure formation demarcated by the color of the initial clone. Within these structures, Lgr5^+^ cells gave rise to Lgr5^−^ cells. This clearly indicated self-renewal and differentiation capacities of Lgr5^+^ CSCs. Re-xenotransplantation showed long-term CSC function [15]. To test the need for Lgr5^+^ CSCs in these processes, they inserted a transgene expressing iCaspase9 into the Lgr5 allele, and upon injection of a Caspase 9 dimerizer compound, triggered apoptosis exclusively in Lgr5^+^ cells. This led to a reduction in tumor size. However, a few days after treatment, Lgr5^+^ cells reappeared and tumor growth resumed, indicating a plastic process [15].

A similar approach using organoids established from a panel of human colorectal tumors was undertaken in parallel by Cortina and colleagues [14]. Lgr5-eGFP patient-derived organoids (PDOs) were implanted into NOD/SCID mice. The xenografts displayed glandular organization and stromal recruitment. Subsequent sorting of cells from the xenograft tumors showed that Lgr5-eGFP^+^ cells from xenografts exhibited a significantly higher clonogenic potential than Lgr5-eGFP^−^ in vitro, and were more tumorigenic upon transplantation in vivo, mimicking the conventional approach to cancer stemness. An elegant color switch strategy allowed them to trace the fate of Lgr5^+^ cells in xenotransplants. They observed heterogeneous growth dynamics: some clones expanded constantly, while others divided slowly or even remained as individual cells over prolonged periods of time [14]. Cortina and colleagues also developed a strategy to evaluate proliferation rates of clones with different differentiation status. They generated Lgr5-eGFP (L) patient tumor organoids that expressed TagRFP fused to the endogenous Ki67 protein (K). When they analyzed the xenografts, Lgr5-eGFP^+^/Ki67-TagRFP^−^ (L+K−) ranged from 20% to 50% of the clones. FACS analysis after xenograft dissociation showed that L-/K-cells displayed downregulation of proliferation genes, upregulation of the cell cycle inhibitor CDKN1A, and expression of markers of terminal differentiation, suggesting that they were terminally differentiated postmitotic cells. L−/K+ cells displayed low levels of stem cell markers and upregulated genes characteristic of early absorptive differentiation, suggesting that they could be in a state analogous to transit-amplifying cells. L+/K− cells showed downregulation of proliferative genes and retained elevated levels of intestinal stem cell markers [14]. Interestingly, these results resemble those of Kreso and colleagues, whereby genetically homogeneous clones unbiasedly labeled by lentiviral transduction showed different proliferation rates along serial transplantation into NSG mice and after chemotherapy [65] (see below in Section 4.2.1).

In summary, lineage tracing methods combined with tumor organoid technologies have not only demonstrated a hierarchical cell organization in relevant colorectal cancer mouse models and in human samples but have also shown that gene expression profiles of CSCs are shared with normal intestinal stem cells. However, in spite of its immense utility, this approach is not exempt from limitations, given mainly by the lack of mesenchymal tissue or tumor stroma and its common culture on Matrigel^TM^, which can potentially introduce uncontrolled variables [66,67]. Although tumor organoids have been successfully kept in minimal culture medium, addition of growth factors boost their development, underscoring the importance of studying the stromal contribution to stem cell function. Nevertheless, these technologies have set a baseline to understand stem cell functions during CRC progression. On this ground, the contribution of differentiated cells to the CSC function and tumor progression can be empirically studied.

## 4. Contribution of Differentiated Cancer Cells to Tumor Progression

### 4.1. Cancer Cell Plasticity

**Figure 3:**
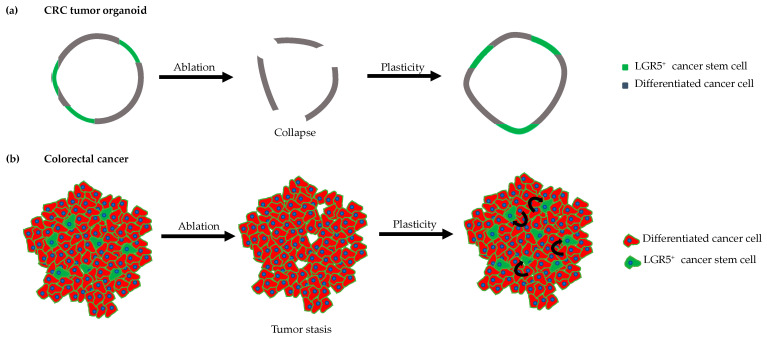
Cancer stem cells (CSCs) are a minority of the tumor mass. (**a**) The ablation of CSCs ex vivo results in a collapse of the tumor organoid. Plasticity enables differentiated cancer cells to become CSCs. (**b**) In an in vivo setting, the ablation of CSCs led to tumor stasis and the reoccurrence of CSCs derived from differentiated cancer cells.

Plasticity is the ability of cells to transition from a differentiated state into an undifferentiated stemness state. After damage to Lgr5^+^ stem cells, the intestinal epithelium exhibits great plasticity [68], and several Lgr5^−^ cells, including fully differentiated cells of secretory lineages, can contribute to replenishing the stem cell compartment after it has been damaged [50,69,70,71,72,73,74] (Figure 3). Below, we assess the importance of this phenomenon for cancer development based on cell hierarchies within tumors.

#### 4.1.1. Signaling Pathways Involved in Cancer Cell Plasticity

How the plastic process is orchestrated in colorectal tumors and which factors are involved is currently unknown. Accumulating evidence shows that extracellular signals from microenvironments within the tumor can exert a spatiotemporal regulation on the cell states and their contribution to tumor growth [75,76]. Lenos and collaborators showed that the invasive front of the tumor, which is in close contact to the surrounding microenvironment, generates a large number of functional stem cells. Through lineage tracing in xenograft tumors from patient-derived engineered colorectal cancer organoids, they showed that clonogenic activity of CSCs takes place at the periphery of tumors, while the central area remains quiescent. Based on a mathematical model, they concluded that this is due to microenvironmental influence in areas of contact between cancer cells and the recruited host stroma, and that osteopontin (OPN) is a key signal coming from surrounding cancer-associated fibroblasts (CAFs) [75]. Using multicolor lineage tracing, van der Heijden and collaborators reported similar spatiotemporal clonogenic dynamics, with small clones in the center of the tumors and expanding clones in the periphery, being the major contributors to tumor growth in response to microenvironmental influences [76]. Relevant to stem cell functionality, the activity of the Wnt signaling pathway itself was shown to be highly dependent on the surrounding environment and to display a similar pattern: high activity at the invasive front of tumors and low activity in the central tumor areas, independent of constitutive Wnt-activating mutations [41,49]. The latter authors identified hepatocyte growth factor (HGF) as a key secreted factor from tumor-stromal myofibroblasts. 

Interestingly, OPN is a ligand of CD44, which is one of the first most commonly used markers of colorectal CSCs [5]. Besides functioning as a cell adhesion molecule, CD44 also functions as a pleiotropic co-receptor, regulating the signal transduction of several signaling pathways simultaneously [77]. Relevant for colorectal cancer, CD44 not only acts as a positive regulator of the Wnt pathway at the level of the signalosome, but is also a Wnt target gene ([78]. Furthermore, effective signal transduction upon HGF binding to its cognate receptor tyrosine-kinase MET requires the co-receptor function of CD44v6 [79,80,81]. Todaro and collaborators showed that CD44v6 is expressed by colorectal CSCs, thus demarcating a clonogenic population, and that OPN, HGF, and CXCL12 secreted by associated colorectal tumor stroma induce expression of CD44v6 through Wnt, priming cells with metastatic potential. Moreover, low expression of CD44v6 in tumor biopsies was significantly associated with prolonged overall survival of patients with stage III and stage IV colorectal cancer [40].

CAFs also induce the Notch signaling pathway, which is involved in regulating stemness in both normal intestinal tissue and in colorectal cancer. Notch inhibition decreases stemness and enhances goblet-like differentiation at all stages of colorectal cancer [82]. Of note, overexpression of active Notch 1 increases the EMT/stemness-associated markers CD44, Slug, and Smad-3 and induces Jagged expression [83]. Notch was also found to be important for colorectal cancer metastasis [84,85]. Recent evidence links Notch signaling with the crypt formation in intestinal organoids. The Liberali group thoroughly dissected cell decision-making during organoid formation from single cells. The signaling involved an uneven nuclear YAP (yes-associated protein) localization in cells differentiating into Paneth cells. These cells expressed DLL1 (Delta-like protein 1) to produce lateral inhibition through Notch, enabling symmetry breaking, crypt formation, and induction of LGR5 expression [86,87].This evidence underscores the potential role of the Notch pathway in cell plasticity. Interestingly, Yap overexpression inhibited Wnt, induced loss of cancer stem cells, and provoked tumor regression in a CRC model [88]. How this relates to Notch signaling is still to be explored.

The RAS/MAPK pathway, which is affected in the adenoma-carcinoma sequence of colorectal cancer, also influences the differentiation state and proliferation of cancer cells and seems to be involved in plasticity. In a screening study of small molecules, Zhan et al. discovered that Mek1 inhibitors are potential activators of the Wnt signaling pathway [89]. Inhibition of the Ras signaling pathway resulted in strong reduction of the transcription factor EGR1 (early growth response protein 1), which is known to regulate the expression of Axin1 by binding to its enhancer region. However, blocking the Ras pathway not only led to upregulation of the Wnt signaling pathway, but also to increased stemness in human tumor organoids, thus indicating reprogramming of differentiated cell types towards a stem cell state. The co-suppression of Wnt and Ras signaling resulted in an increase in differentiated cells and reduced expression of stem cell markers, suggesting that Wnt and Ras signaling have a role in plasticity of colorectal cancer. 

#### 4.1.2. Plasticity to Overcome Metastatic Selective Bottlenecks: Differentiation States as an Adaptive Trait 

Along their journey from the primary tumor to the formation of metastases, cancer cells pass through diverse challenging environments in a multistep process called the invasion-metastasis cascade [90]. The vast majority of bloodborne cancer cells do not succeed in establishing distant organ metastases [90,91,92,93]. Successive challenges like anoikis, stress by shear forces in circulation, immune surveillance by natural killer cells, ability to arrest and adhere to the endothelium, and migration into the parenchyma of target organs impose high selective pressures on migrating cancer cells. Colorectal cancer models have demonstrated the need for Lgr5^+^ CSCs to initiate and maintain full-blown metastasis [13]. However, these observations shed no light upon the previous steps undergone by the colonizing cancer cells. To this end, Fumagalli and collaborators observed these processes through multiphoton intravital microscopy in a spontaneous metastasis model after tumor organoid orthotopic implantation. They found that both Lgr5^+^ and Lgr5^−^ cells escaped from the primary tumor, but that a significant majority were Lgr5^−^ cells. These cells appeared to achieve slightly higher displacement velocities compared to Lgr5^+^ cells. When they analyzed bloodborne cancer cells from primary tumors or pre-established metastases, they found that nearly all circulating tumor cells were Lgr5^−^ cells. All disseminated cells found in the liver as single cells were also Lgr5^−^ cells. Only after the seeded Lgr5^−^ cells developed into lesions over a certain size threshold did they observe appearance of Lgr5^+^ stem cells in all metastatic lesions [16]. Although these experiments do not exclude the possibility that Lgr5^+^ bloodborne cells lost their Lgr5 expression in the absence of a supportive niche, they still show that the metastases were seeded by Lgr5^−^ cells. Complemented by ex vivo tumor organoid experiments, this study show that Lgr5^−^ colorectal cancer cells have a basal ability to give rise to Lgr5^+^ cells, and that this process can be boosted by supportive-niche factors like HGF and FGF [16]. In summary, differentiation and dedifferentiation processes both appear necessary to overcome the challenges faced by colorectal cancer cells during progression to metastatic disease. 

### 4.2. Selective Advantages of Cancer Cell Phenotypic Heterogeneity

#### 4.2.1. Variable Selection of Clones with Distinct Phenotypic Traits

Genetic diversity between cancer cellular clones is source of heterogeneity within tumors [94]. However, there are also non-genetic sources of variability within clones. These correspond either to epigenetic patterns or phenotypes induced by microenvironmental influence [2,10]. To determine the extent to which this variability influences tumorigenicity and its implications in response to cancer therapy, Kreso and collaborators used lentivirally transduced cellular labels to unbiasedly trace the dynamics of different clones from 42 human primary colorectal cancers. They prospectively followed the clonal composition during serial transplantation into immunocompromised mice [65]. Deep sequencing of mutational hotspots of single cells revealed a certain genetic homogeneity within the xenografts and stability along the transplants. However, they noted important differences in cell behavior. Some clones were persistently observed during all passages, while others that were initially apparent became undetectable for a number of passages but reappeared at later points [65]. In a similar approach, Dieter and collaborators found three distinct tumor initiating cell populations in xenografts of lentivirally labeled patient-derived colorectal tumor cells: An extensively self-renewing long-term population able to recapitulate tumor formation upon serial transplantation, a transient amplifying with scarce self-renewal capacity, contributing to tumor formation only in primary mice, and a rare delayed contributing population, only active in secondary and tertiary mice [95]. These marker-free experiences, though not proving that the phenotypical cell heterogeneity found was derived from cell hierarchies due to differentiation, argue in favor of a survival advantage of tumors composed of distinct populations with varying cell behaviors.

#### 4.2.2. Clonal Cooperation

Phenotypic heterogeneity does not only confer selective advantages to specific clones but can also establish cooperative relationships between cells of distinct differentiation fates. Challenging the assumption that carcinogenesis involves a breakdown in cell–cell cooperation and that different clones in a tumor become self-interested competitors, Cleary and collaborators explored the possibility of clonal cooperation within tumors [96]. They used a breast cancer mouse model overexpressing Wnt1, which induces tumor initiation by mammary epithelial progenitor cells. These cells can differentiate into luminal and basal tumor cell types. Since only luminal cells produce Wnt1, the basal cells depend on them to proliferate. Both subclones were required for efficient tumor propagation [96].

In an approach specifically designed to assess intra-tumoral clonal cooperation, under microenvironmental constraints, Marusyk and collaborators analyzed a panel of 18 subclones of the human breast cancer cell line MDA-MB-468 implanted into the mammary fat pad of immunodeficient *Foxn1*nu mice. Using pools of transduced cells rather than clones derived from single cells, they compared every subclone competing with all other subclones (polyclonal tumor). They found that tumor growth could be driven by a proportionally minor subpopulation, which secreted factors that enhanced the proliferation of other subclones. Breakage of this cooperative equilibrium due to a predominant, “excessively fit” clone that outcompeted the cooperative minority provoked tumor collapse and regression. Using a mathematical model applied to non-cell autonomous-driven tumors, they reported that clonal interference, which restrains the excessive predominance of a highly proliferative clone, can stabilize sub-clonal heterogeneity, thus maintaining the sub-clonal interactions necessary for tumor development and evolution of new traits [97]. 

These experimental models show that non-cell autonomous relationships—through direct paracrine action or through microenvironmental regulation—between cancer cell subclones may be essential for tumor development. Although they do not show that the involved cells are derived from differentiating hierarchies, they suggest that tumorigenicity is not exclusively driven by a certain cell type. Whether the phenotypic heterogeneity derived from cell hierarchies in colorectal cancers can entail selective advantages of subclones or cooperative relationships is still to be explored.

#### 4.2.3. The Role of Hierarchical Cancer Cells’ Organization in Therapeutic Resistance

Recapitulation of tumors’ structures upon recurrence after chemotherapeutic treatment have placed CSCs at the core of drug resistance. Even when chemotherapy has reached a high level of efficiency, a small population of cancer cells may still be resistant, which is often referred to as minimal residual disease [98]. This is believed to be due to CSCs’ similarities with normal stem cells such as quiescence, efficient DNA repair, and multidrug resistance (MDR) due to high expression of ABC transporters [99,100]. Lgr5^+^ cancer stem cells not only showed the typical stem cell signature, but also a reduction in cell-cycle-related signatures, supporting their potential function in chemotherapy resistance [101]. Putative CSCs had been shown to express high levels of ABC transporters and ROS (reactive oxygen species) decrease by ALDH (aldehyde dehydrogenase), making them less sensitive to cytotoxic chemotherapeutic drugs [102,103].

Soon after the extension of the hypothesis of CSCs into carcinomas, and the development of panels of markers to identify this cell subpopulation in various cancers, an empirical link was established between this phenotype and the induction of epithelial-to-mesenchymal transitions (EMT) in breast cancer cells [104]. EMT, a process known from the cell rearrangements during embryonic development, can be induced either by ectopic expression of one of several transcription factors (EMT-TFs: Snail, Slug, Zeb1-2, Twist 1-2, and TCF3) or by extracellular signals like TGF-β1 [105]. This leads to the induction of a stem-cell-like phenotype based on surface markers, an enhanced ability to form mammospheres in vitro, and tumor initiation in vivo [104]. The role of this cellular program, however, remains controversial during tumor progression in vivo, being only partially induced with non-redundant effects of the various EMT-TFs [106]. Mesenchymal states confer several survival traits, like decreased apoptosis signaling, increased drug efflux, proliferative quiescence, increasing resistance to conventional cytotoxic or cytostatic therapies, while circumventing molecular-targeted inhibition and desensitizing to immunotherapies based on dendritic cells or immune checkpoints [105].

The notion that CSCs confer resistance to anticancer therapies is supported by the observation that tumors that have been treated with chemotherapy can develop an increased population of CSCs. This could be due to the enhanced survival of the compartment of CSCs. However, the idea that EMTs induce stem-cell-like phenotypes from epithelial cells in a differentiated state also implies a mechanism of cancer cell plasticity. Since cytotoxic chemotherapeutic agents can induce EMTs in cancer cells, the accumulation of CSCs after therapy could be due to differentiated cells constantly feeding this compartment through EMTs [107].

Nevertheless, therapeutic resistance is not necessarily restricted to CSCs. Asfaha and collaborators showed that in the intestinal crypt, Lgr5^−^/keratin-19^+^ (Krt19^+^) progenitor cells mark radio-resistant cells above the crypt. These cells were able to dedifferentiate into Lgr5^+^ intestinal stem cells, replenishing the pool of intestinal stem cells damaged by radiation. When they specifically targeted Lgr5^−^/Krt19^+^ cells with an inducible APC homozygous mutation, these cells initiated autochthonous malignant tumors containing mutated Lgr5^+^ cells—therefore generated through cell dedifferentiation—that drove tumor development. Irradiation of these mice 24 h after induction of this mutation did not prevent tumor development and rapid mortality. In contrast, tumors initiated by specifically inducing the same APC mutation in Lgr5^+^/Krt19^−^ intestinal stem cells did not provoke mortality in mice irradiated 24 h after the mutation induction [71]. This meant that mutated intestinal stem cells (Lgr5^+^) were radiosensitive, while non-stem cell tumor-initiating clones were radio-resistant. Part of the progeny of the latter subsequently dedifferentiated into Lgr5^+^ cells and reestablished a hierarchical organization to drive tumor development.

In tumors such as colorectal cancers with clear cellular hierarchies, independently of which cellular state is more prone to resist therapy, the possibility of phenotypic switches from differentiated cells to stem cells appears fundamental to tumor relapse. This plasticity seems therefore an essential consideration for therapeutic development.

## 5. Conclusions

From a Darwinian-selection perspective of cancer progression, cell hierarchies add a third source of cancer cell fitness to the one derived from genetically fixed and epigenetically stable acquired traits. Plasticity between cell differentiation states ensures adaptability to selective pressures. In this scenario, selective advantages of cancer cells derive not only from genetic or epigenetic makeups that deploy specific hallmarks of cancer, but also from the ability to dynamically deploy different hallmarks that ensure better fitness in changing environments. Cancer cells can self-renew in microenvironments that are permissive enough to drive tumor progression and give rise to differentiated cells endorsed with specific properties. In turn, differentiated cells can also dedifferentiate, giving rise to CSC when required. This enables cancer cells to either contribute to shaping these microenvironments—in accordance with tumor sub-clonal cooperation—or to overcome a plethora of diverse and dynamic selective pressures. 

In conclusion, the notion of plasticity between cellular states not only supports the CSC hypothesis in colorectal cancers, but also underscores a role for the differentiated states of tumor cells during cancer progression. This expands the scope of therapeutic development beyond stemness to include other cellular functions, amongst which cell plasticity itself is beginning to emerge as the next target.
